# Exploring cellular uptake, accumulation and mechanism of action of a cationic Ru-based nanosystem in human preclinical models of breast cancer

**DOI:** 10.1038/s41598-019-43411-3

**Published:** 2019-05-07

**Authors:** Marialuisa Piccolo, Gabriella Misso, Maria Grazia Ferraro, Claudia Riccardi, Antonella Capuozzo, Mayra Rachele Zarone, Francesco Maione, Marco Trifuoggi, Paola Stiuso, Gerardino D’Errico, Michele Caraglia, Luigi Paduano, Daniela Montesarchio, Carlo Irace, Rita Santamaria

**Affiliations:** 1 Department of Pharmacy, University of Naples “Federico II”, Via D. Montesano 49, 80131 Naples, Italy; 2 Department of Precision Medicine, University of Campania “Luigi Vanvitelli”, Via L. De Crecchio 7, 80138 Naples, Italy; 3 Department of Chemical Sciences, University of Naples “Federico II”, Via Cintia 21, 80126 Naples, Italy; 4 CSGI - Consorzio Sistemi a Grande Interfase, Department of Chemistry, University of Florence, Via della Lastruccia 3, 50019 Sesto Fiorentino (FI), Italy

**Keywords:** Breast cancer, Autophagy, Drug delivery

## Abstract

According to WHO, breast cancer incidence is increasing so that the search for novel chemotherapeutic options is nowadays an essential requirement to fight neoplasm subtypes. By exploring new effective metal-based chemotherapeutic strategies, many ruthenium complexes have been recently proposed as antitumour drugs, showing ability to impact on diverse cellular targets. In the framework of different molecular pathways leading to cell death in human models of breast cancer, here we demonstrate autophagy involvement behind the antiproliferative action of a ruthenium(III)-complex incorporated into a cationic nanosystem (HoThyRu/DOTAP), proved to be hitherto one of the most effective within the suite of nucleolipidic formulations we have developed for the *in vivo* transport of anticancer ruthenium(III)-based drugs. Indeed, evidences are implicating autophagy in both cancer development and therapy, and anticancer interventions endowed with the ability to trigger this biological response are currently considered attractive oncotherapeutic approaches. Moreover, crosstalk between apoptosis and autophagy, regulated by finely tuned metallo-chemotherapeutics, may provide novel opportunities for future improvement of cancer treatment. Following this line, our *in vitro* and *in vivo* preclinical investigations suggest that an original strategy based on suitable formulations of ruthenium(III)-complexes, inducing sustained cell death, could open new opportunities for breast cancer treatment, including the highly aggressive triple-negative subtype.

## Introduction

Breast cancer is the second most common cancer worldwide after lung cancer, the fifth most common cause of cancer death, and the leading cause of cancer death in women^1,2^. According to the World Health Organization (WHO), its incidence is increasing so that novel chemotherapeutic options are nowadays essential in order to kill specific cancer types, as well as to overcome both non-cancer cell toxicity and treatment failure due to chemoresistance^3^. Despite these alarming data, in the last decades death rates from some cancer types have sensibly decreased, and this is due - at least in part - to the advances of chemotherapy^4,5^. The heterogeneity and variety of breast cancer subtypes make them a demanding solid tumour to diagnose and treat, with multiple possible drug targets to exploit in developing effective therapies^6,7^. In this context, the activation of multiple death pathways in cancer cells throughout the tuning of new metal-based chemotherapeutics represents one of the main current objectives^8,9^. Metal complexes have always held great potential as anticancer agents against a wide majority of cancer types^10,11^. In the design of innovative metal-based chemotherapeutics, numerous ruthenium complexes have been lately screened against a number of cancer cell lines, showing enhanced selectivity for breast cancer cells with reduced side effects on healthy cells^12–14^. Beyond preclinical investigations and due to their unique biochemical properties, they are gaining importance in clinic because of lower toxicity and more specific activity than many current metallo-drugs^15^. In this frame, NAMI-A and KP1019 are noteworthy being the first ruthenium(III)-based anticancer drugs which entered clinical trials (Fig. S1a,b)^16,17^. As well, many other ruthenium-containing compounds have been developed and tested as drug candidates, both inorganic and organometallic^18^. Thermodynamic and kinetic stability of Ru(III) complexes, as well as their bioactivity, are generally lower than that of Ru(II) complexes. Hence, behaving as prodrugs of Ru(II) complexes, many Ru(III) compounds contain exchangeable ligands and require activation by reduction in the tumour microenvironment^19,20^. In addition, many nanomaterial Ru complexes have been recently designed and developed into anticancer drugs with interesting beneficial properties^21,22^. In this context, a well-characterized mini-library of highly functionalized Ru(III) nanosystems designed by our group, was screened in preclinical evaluations on several cancer cells from solid tumours, showing attractive bioactivity profiles endowed with high antiproliferative effects^9,23^. In particular, to improve the Ru complexes efficacy as anticancer drugs in biomedical applications, we have utilized nucleolipid-based platforms^24–26^ to develop original nanosystems capable of deliver an antiproliferative Ru(III) complex, named AziRu (Fig. S1c) and inspired to the well-known NAMI-A. The ultimate formulations used in bioscreens were obtained by co-aggregation of the nucleolipid Ru(III)-complexes with zwitterionic or cationic lipids, providing stable and biocompatible liposome formulations for cancer therapy^27–31^. The most promising outcomes were achieved on human breast models *in vitro*, including the epithelial-like estrogen-dependent breast adenocarcinoma MCF-7 cell model, and the mesenchymal-like triple-negative breast adenocarcinoma MDA-MB-231 cells, accounting for the great majority of investigations on breast cancer cells (BCC)^9^. Accordingly, these BCC lines are likely to reflect, to a large extent, the features of cancer cells *in vivo*^32^.

In the context of a promising ruthenotherapy and allowing for the elucidation of the mode of action of anticancer Ru(III)-based drugs, we previously reported an impression of the molecular mechanisms underlying the biological effects of our formulations. Our research revealed that apoptotic pathways were involved in the action mechanisms of the Ru(III) complex AziRu, inducing cancer cell death^9^. Following this path, here we provide new insights into the effectiveness of our formulations in human breast cancer models, focusing on ruthenium accumulation in specific subcellular compartments subsequent to nanosystems administration *in vitro*, and on its ability to induce an additional pathway of programmed cell death, *i*.*e*. autophagy. This mechanism of cell death, associated with the appearance of autolysosomes, is dependent on the coordinated activity of autophagy-related (Atg) proteins^33^, orchestrating a vital process for defence against disease to maintain cellular homeostasis^34^. Indeed, specific stimuli can activate selective autophagic pathways to address specific stressor^35^. The role of autophagy in cancer has been vastly investigated and reviewed; nowadays evidence unravel the role of autophagy either as tumour suppressor or in tumour cell survival depending on several dynamics^36^. Its dual role is further confused by specific cancer microenvironments and makes autophagy induction a very complex process to be exploited in response to treatments^37,38^. As well, some Ru(II) complexes have proven to be capable of activating autophagy in cancer cells, though occasionally in antagonism to mitochondria-mediated apoptosis^39,40^. For sure, the activation of autophagic pathways by chemotherapeutics deserves further investigations, and represents another challenging possible molecular mechanism in order to inhibit uncontrolled proliferation of cancer cells^41^.

**Figure 1:**
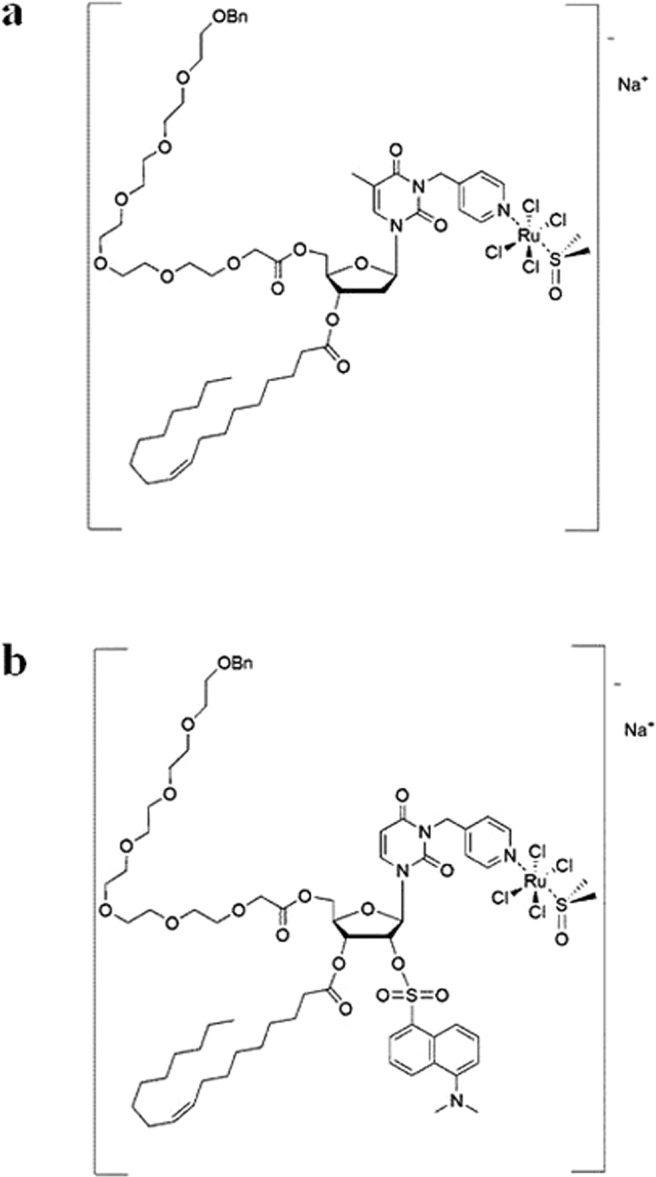
Molecular structures of the anticancer ruthenium(III)-complexes. (**a**) The Ru(III) complex HoThyRu, and (**b**) the fluorescently labeled Ru(III) complex HoThyDansRu (Bn = benzyl).

In support of this study, we have benefited from using a cationic DOTAP-based nanosystem loaded with the nucleolipid Ru(III)-complex HoThyRu, providing the HoThyRu/DOTAP formulation (see Fig. 1a), hitherto one of the most effective among the nucleolipidic nanosystems we have designed for the *in vivo* transport of the AziRu complex. This liposomal system contains 15% in moles of the nucleolipidic complex HoThyRu, which at this composition is stable for several months^30^. Here, by an *ad hoc* designed fluorescent analogue of the nucleolipidic Ru(III)-complex co-aggregated with the same lipid DOTAP, named HoThyDansRu/DOTAP (Fig. 1b), and confocal microscopy approach, in concert with subcellular fractionation and inductively coupled plasma-mass spectrometry (ICP-MS) analysis, we have thoroughly explored ruthenium trafficking in BCC. The action of HoThyRu/DOTAP formulation as autophagy-inducer agent was further investigated in the context of preclinical trials, thus proposing an innovative strategy for anticancer therapy based on the concurrent activation of multiple cell death pathways. In addition, by a xenograft model of human BCC we have validated the efficacy and the safety *in vivo* of our ruthenium-based candidate drugs in the perspective of novel cancer therapeutic options.

## Results

### Antiproliferative efficacy of the HoThyRu/DOTAP nanosystem on breast cancer cells *in vitro*

The antiproliferative efficacy of HoThyRu/DOTAP formulation against MCF-7 and MDA-MB-231 cells was examined by mitochondrial metabolic activity and cell counting assays, grouping data into a single indicator defined as cell survival index^31^. In Table 1 the resulting IC_50_ values are shown, including those of cisplatin and of the Ru(III)-complex AziRu, herein used as reference drugs. In accordance with our recent reports, HoThyRu incorporated into the DOTAP nanocarrier exhibited marked cytotoxicity *vs* breast cancer cell lines, whereas the “naked” AziRu was almost inactive under the same experimental conditions. Many studies in recent years by our group have indicated that this approach - in which we essentially decorate the AziRu core with a nucleolipid and insert it into a lipid carrier - allows converting a non-antiproliferative compound (AziRu showed a behaviour very similar to NAMI-A, which is antimetastatic and is not antiproliferative) into a cytotoxic species, selectively active on cancer cells. Among the nucleolipidic ruthenium complexes we have developed, HoThyRu, particularly when transported by the DOTAP nanosystem, proved to be the most bioactive *in vitro*^9,23^. The structure and properties of the decorated nucleolipid and of the lipid nanocarrier have a major impact on the anticancer activities of the ruthenium core by affecting its *in vivo* transport and stability, cellular uptake, localization and mechanisms of action^9,28,30,31^. Remarkably, in the same experimental conditions this Ru-based nanosystem proved to be even more effective than cisplatin. At the same time, HoThyRu/DOTAP is basically inactive on MCF-10A cells (IC_50_ higher than 250 µM), herein used as a reliable and specific model *in vitro* for normal human mammary epithelial cells. Accordingly, evidences emerging from non-malignant cells, including those we previously reported for human HaCaT keratinocytes and rat L6 myoblasts^31^, suggest a significant selectivity of action of our nanosystems in BCC.

**Table 1:** Anticancer activity of the HoThyRu/DOTAP nanosystem in breast cancer cells.

IC_50_ values (µM) of the HoThyRu/DOTAP nanosystem, AziRu and *c*DDP				
	HoThyRu/DOTAP		AziRu	*c*DDP
BCC lines	Liposome	**Ru complex**		
MCF-7	85 ± 4	**12.7** **±** **4**	>250	17 ± 5
MDA-MB-231	81 ± 3	**12.1** **±** **3**	>250	19 ± 4
MCF 10 A	>250	>**100**	>250	*NA*

IC_50_ values (µM) of the HoThyRu/DOTAP liposomial formulation (Liposome), of the actual nano-delivered Ru complex (**Ru complex**), of the naked AziRu complex (AziRu), and of cisplatin (*c*DDP) here used as cytotoxic reference drug, in MCF-7 and MDA-MB-231 BCC lines, as well as in MCF 10A non-tumourigenic epithelial cell line, after 48 h of incubation *in vitro*. The ruthenium IC_50_ values corresponding to the effective metal concentration (15% mol/mol) carried by each nanoaggregate are shown in bold. IC_50_ values are reported as mean ± SEM (*n* = 30). (*NA* = *Not Assessed*).

### Cellular uptake and localization of HoThyRu/DOTAP

**Figure 2:**
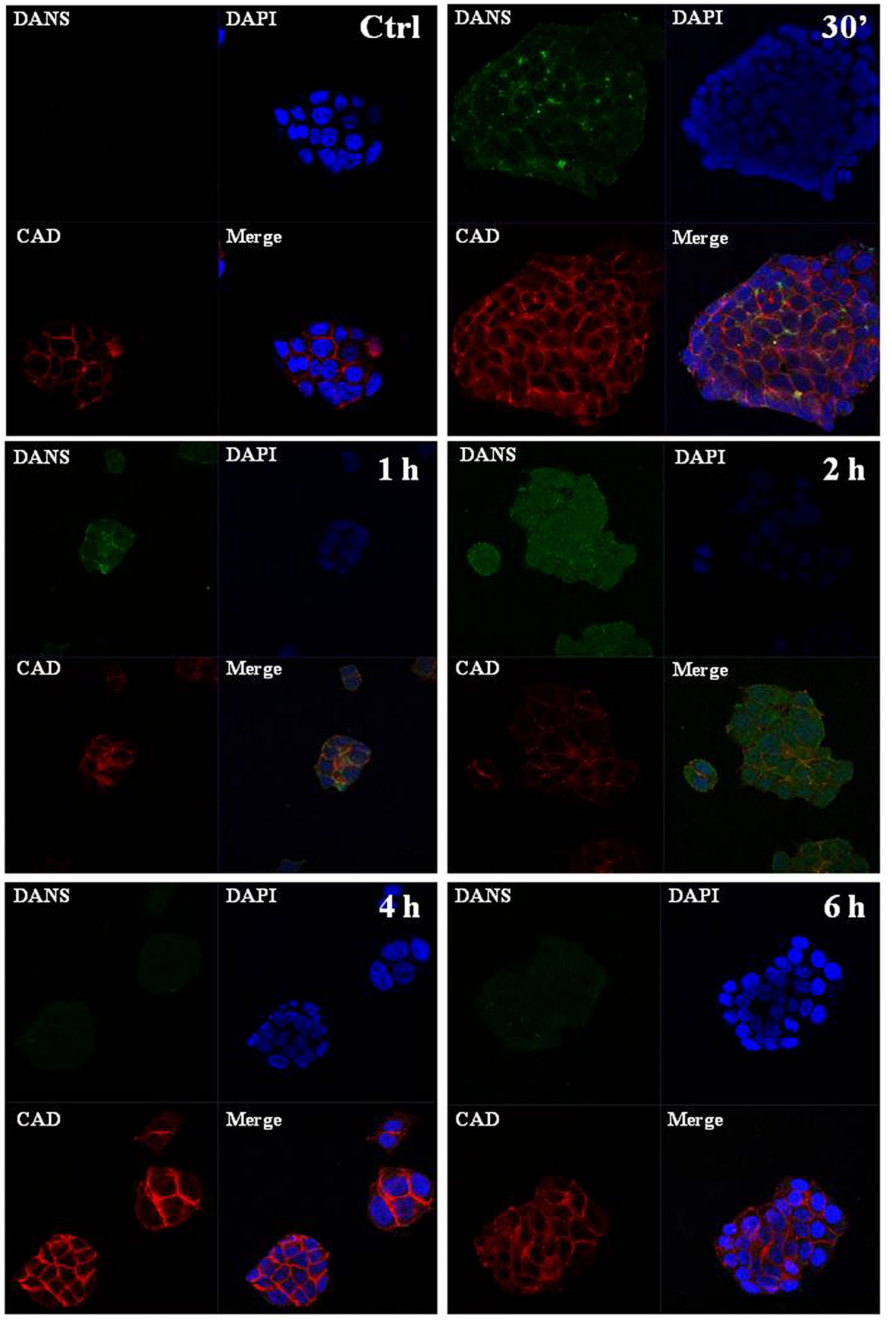
Uptake and localization of the HoThyRu/DOTAP nanosystem in MCF-7 cells. Confocal microscopy bioimaging of cellular uptake of the fluorescent HoThyDansRu/DOTAP nanosystem in MCF-7 cells incubated for 0.5, 1, 2, 4 and 6 h at 100 µM. Nuclei blue fluorescence is due to DAPI (E_x_358, E_m_461 nm). Green dots within cells represent dansyl-associated fluorescent emission (DANS) related to HoThyDansRu/DOTAP (E_x_370, E_m_450–550 nm). E-cadherine-associated red fluorescence (CAD) defines cell shape (E_x_650, E_m_668 nm). In merged images (Merge), the fluorescent patterns from cell monolayers are overlapped. The shown images are representative of three independent experiments. 100× total magnification (10× objective and a 10× eyepiece).

Cellular uptake plays an important role in drugs bioactivity, so that internalization and accumulation of metal-based drugs into cancer cells is crucial for the therapeutic effect against tumours. For this reason, we have largely investigated nucleolipidic Ru-containing liposomes interactions with cell membranes, along with their cell internalization processes^23^. By means of fluorescent probes loaded into POPC and DOTAP liposomes we have previously demonstrated that nucleolipidic nanoaggregates rapidly interact with biological membranes allowing a massive cellular uptake, even after short incubation times such as 30 min and 1 h^28,30,31^. To give a further insight into the action mechanism of the Ru complex once inside cancer cells and likely interactions with specific biomolecular targets, experiments by confocal microscopy have been carried out. Aiming at determining the subcellular location of the ruthenium complex, the nucleolipidic Ru(III)-complex has been replaced with its analogue equipped with a fluorescent tag, *i*.*e*. the dansyl group, providing the dansyl-labeled nucleolipid Ru(III)-complex co-aggregated with DOTAP, to give the HoThyDansRu/DOTAP nanosystem. In this way, the fate of the active ruthenium complex was directly assessed, thereby examining its location after nanocarriers application to MCF-7 monolayers. In bioimaging, bright green dots within cells represent dansyl-associated fluorescent emission (DANS) produced by the HoThyDansRu complex. Blue fluorescence is due to DAPI, a stain that labels DNA and shows the position of the nuclei. Red E-cadherine-associated fluorescence highlights adherens junctions and cell-cell contacts, since MCF-7 cells grow as epithelial clusters. Merged confocal images are obtained by overlapping fluorophore emissions from the same cell monolayer. Consistently with our previous results, the dansyl-labeled complex localizes rapidly within MCF-7 cells, and confocal microscopic images in Fig. 2 clearly show a time-dependent cellular accumulation. The dansyl-dependent fluorescence occurs after very short contact times (30 min), reaching the fluorescence maximum diffusion after 2 h from the beginning of the treatment. Accordingly, analysis of the dansyl-dependent fluorescence emission over time suggests that the Ru-complex lodged in DOTAP liposomes is first localized at the level of cell membranes, and then enters the cytoplasm, spreading to the whole cell including the perinuclear and nuclear compartments finally. Indeed, after 2 h of incubation the dansyl-dependent green fluorescence emission is detectable also in the nuclei area, although attenuated by DAPI staining in merged images. Overall, in addition to denoting an effective cellular uptake process, the fluorescent patterns seem to suggest an intracellular release of the pharmacologically active agent after liposome degradation, coupled to its cytoplasmatic and nuclear spread. In fact, the extensive and widespread generation of dansyl-associated spots following cellular uptake is consistent with the effective nanocarriers internalization. Remarkably, at longer incubation times (*i*.*e*. 4 and 6 h), a significant decrease in the dansyl-dependent fluorescence signal intensity occurs, supporting the hypothesis that the nanosystem releases the active ruthenium complex within the cell. The fluorescence intensity of the dansyl probe is in fact largely affected by its environment and/or external stimuli. Typically, it exhibits high intensity fluorescence only in apolar media, with large solvent-dependent shifts of the maxima; conversely, when exposed to water, only basal fluorescence is detected^42^. Therefore, the data obtained by monitoring over time the fluorescence intensity of cells treated with the HoThyDansRu/DOTAP nanosystem provide a nice evidence of the efficacy of the lipid nanocarrier, effectively internalizing HoThyDansRu. Accordingly, its fluorescence intensity is initially very marked because the dansyl dye is stably incorporated in the apolar region of the DOTAP lipid bilayer, and progressively less and less intense as the nucleolipid Ru-complex is released in the intracellular media.

### Sub-cellular accumulation of Ru(III)-complexes

**Figure 3:**
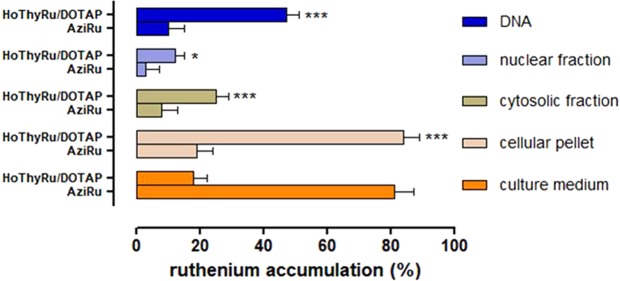
Cellular and sub-cellular Ru(III)-complex trafficking after HoThyRu/DOTAP application to MCF-7. Ruthenium distribution between MCF-7 cells and media, plus intracellular ruthenium accumulation in cells by subcellular fractionation and inductively coupled plasma-mass spectrometry (ICP-MS) analysis, after incubations for 24 h with 100 µM of AziRu or HoThyRu/DOTAP formulation. In the reported fractions, ruthenium content is expressed in bar graphs as percentage of the total ruthenium administered during incubations *in vitro*. Results derive from the average ± SEM values of three independent experiments. *p < 0.05 *vs*. AziRu treated cells; ***p < 0.001 *vs*. AziRu treated cells.

The results on the Ru complex intracellular and metabolic fate next to HoThyRu/DOTAP application to cells were further supported by subcellular fractionation performed on MCF-7 cells, in combination with inductively coupled plasma-mass spectrometry (ICP-MS) analysis. As shown in the bar graph in Fig. 3, ruthenium assessment and localization after treatment *in vitro* for 24 h with HoThyRu/DOTAP (100 µM) provide clear evidence that cellular uptake is considerably increased by the nanoformulation with respect to treatments carried out with the “naked” AziRu complex (100 µM). Indeed, while a large extent of AziRu remains in the culture medium after incubation (about 80%), large amounts of ruthenium (about 85% of the administered quantity) are found at cellular level after treatment with HoThyRu/DOTAP liposomes. Moreover, ICP-MS analysis performed on the isolated subcellular fractions indicates that the liposomal ruthenium portion entering the cells is broadly distributed amongst the intracellular compartments, but above all at the nuclear level as evidenced by the high metal content bound to nuclear DNA (virtually almost 50% of the all liposomal AziRu administered during treatment *in vitro*). Obviously, under the same experimental conditions, also AziRu administered as such is distributed within the cell, but to a lesser overall extent given its limited cellular uptake. In conclusion, ruthenium accumulation in cells and subcellular compartments is in all cases quantitatively greater when cells are treated with HoThyRu/DOTAP formulation compared to the “naked” AziRu. These data are in total agreement with confocal microscopy experiments.

### Autophagy detection

**Figure 4:**
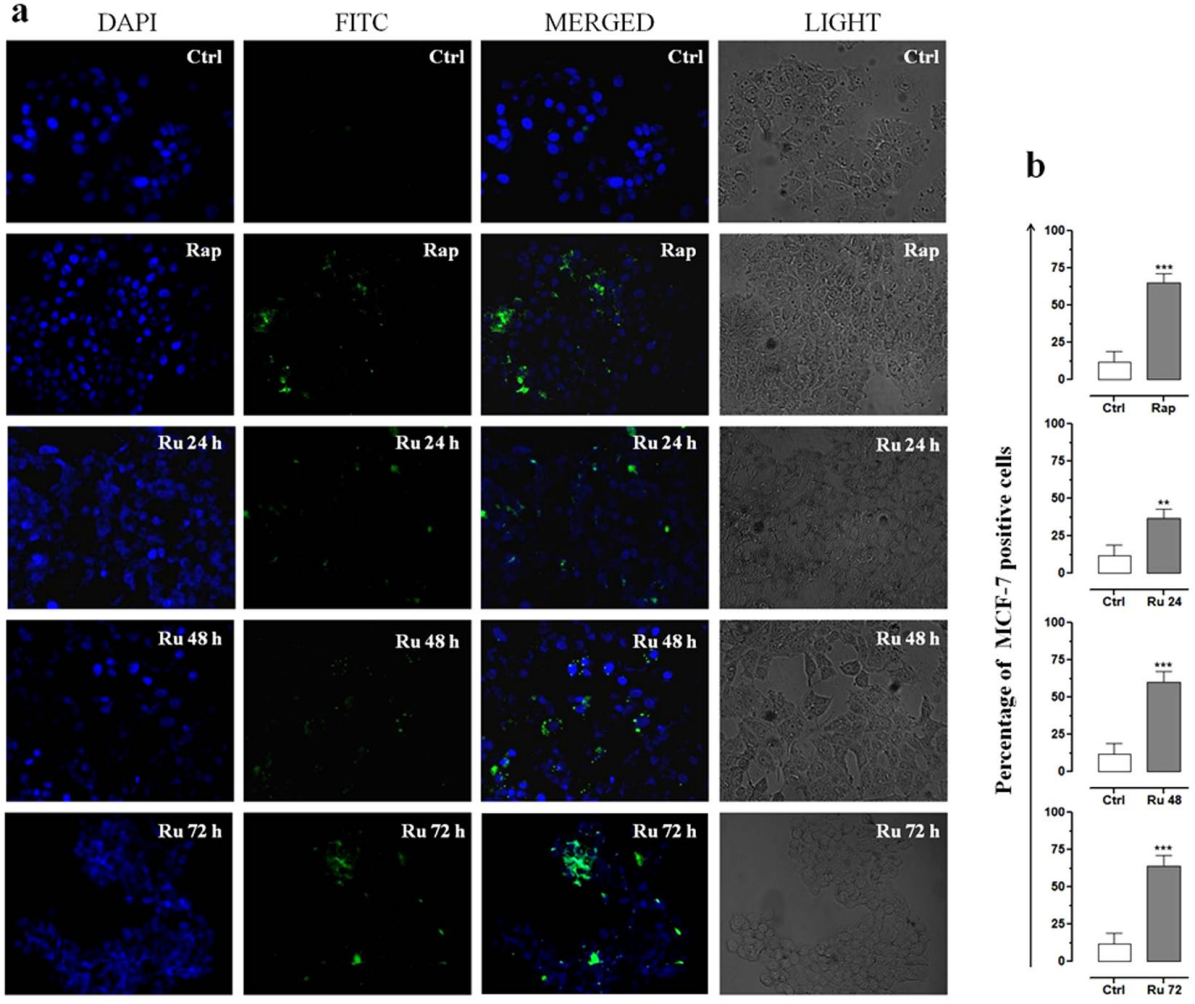
Autophagy detection in MCF-7 cells. (**a**) Autophagy detection by fluorescent microscopy analysis showing nuclei (blue nuclear stain, DAPI filter) and autophagic vesicles (green, FITC filter) in control MCF-7 cells (Ctrl), or in cells treated with 10 µM rapamycin (Rap) for 48 h, and with IC_50_ of HoThyRu/DOTAP for 24, 48, and 72 h (Ru 24 h, Ru 48 h, and Ru 72 h, respectively). In merged images (MERGED), the fluorescent patterns from cell monolayers are overlapped. In addition, phase-contrast light microphotographs (LIGHT) at 600× magnification (30× objective and a 20× eyepiece) of MCF-7 cells treated or not with rapamycin and HoThyRu/DOTAP as indicated before, are shown for detection of both cellular morphological changes and cytosolic autophagic vesicles formation. The displayed images are representative of three independent experiments. (**b**) Percentage of Green Detection Reagent-positive MCF-7 cells following the indicated treatments *in vitro* with respect to untreated control cells. **p < 0.01 *vs*. control cells; ***p < 0.001 *vs*. control cells.

In eukaryotic cells, autophagy is considered a dynamic multi-step process, regulated both positively and negatively at several points. Autophagic activity is typically low under basal conditions; however it can be evidently up-regulated both in whole living organisms and in cultured cells by a variety of physiological and non-physiological stimuli, including pharmacological treatments *in vitro*^35,36^. In order to monitor the autophagic flux in breast cancer cells following HoThyRu/DOTAP liposome application *in vitro*, MCF-7 and MDA-MB-231 cells were processed by a fluorescent Autophagic Detection Kit. In this way fluorescent microscopy analysis allows showing nuclei (blue nuclear stain, DAPI filter) and autophagic vesicles, such as autophagosomes and autophagolysosomes (green perinuclear and cytosolic stain, FITC filter), as shown in Fig. 4 for MCF-7 cells. There are several approaches known to induce autophagy *in vitro*. Rapamycin, one of the most potent recognized inducer of autophagy, is herein used as positive control^43^. Since response to rapamycin is time- and concentration-dependent, and may also significantly vary depending upon cell type and cell line, we performed a preliminary study to fine-tune the best experimental conditions. Accumulation of the green fluorescent probe as detection reagent was particularly evident in MCF-7 treated for 48 h with 10 µM rapamycin, which induces the formation of autophagic vesicles. Then, to evaluate the impact of incubation time on autophagy induction by HoThyRu/DOTAP at the IC_50_ (85 µM, *i*.*e*. 12.7 µM in HoThyRu), experiments were carried out for 24, 48 and 72 h. Merged images by overlapping fluorophore emissions from the same MCF-7 cell monolayer show an important population of Green Detection Reagent-labeled vesicles. By time course microphotographs, HoThyRu/DOTAP induces a progressive increase in punctuate structures matching to autophagic vacuoles, as confirmed by the quantization of the fluorescent signal showing a significant time-dependent increase in the number of positive cells after treatments *in vitro* (Fig. 4b). After 72 h of incubation with HoThyRu/DOTAP at IC_50_, the effect on MCF-7 cells is substantially similar to that of 10 µM rapamycin. Very similar results in terms of fluorescent emission were obtained in MDA-MB-231 cells after analogous treatments (data not shown). Phase-contrast light microscopy imaging at 600× magnification (30× objective and a 20× eyepiece) confirms the ability of HoThyRu/DOTAP to promote a massive formation of autophagic vacuoles detectable in cell cytoplasm. After 72 h of incubation, the green fluorescent emission is widespread in cells and merged images show a strong fluorescence accumulation in spherical vacuoles in the perinuclear region, as well as in foci distributed throughout the cytoplasm.

### Expression profile of autophagy-related proteins after HoThyRu/DOTAP treatment

**Figure 5:**
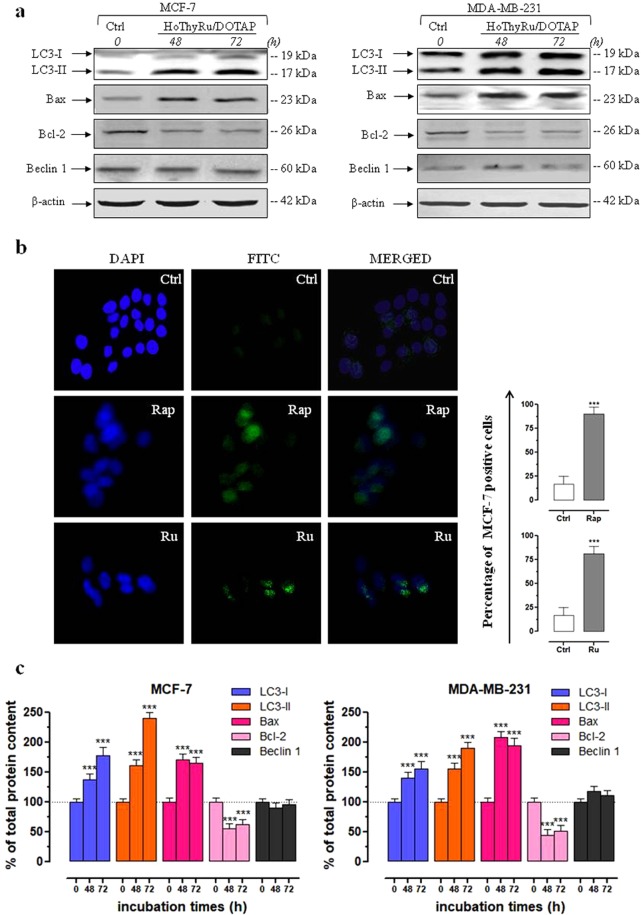
Autophagy-related proteins after HoThyRu/DOTAP treatment in MCF-7 and MDA-MB-231 breast cancer cells. (**a**) Western blot analysis showing the effects of IC_50_ concentrations of HoThyRu/DOTAP following 48 and 72 h of incubation in MCF-7 (left panels) and MDA-MB-231 cells (right panels) on expression of LC3-I and LC3-II, on Bax and Bcl-2, and on Beclin 1, to characterize the autophagic response (Ctrl, untreated cells at the time zero). The shown blots are representative of three independent experiments and are cropped from different parts of the same gel, as explicit by using clear delineation with dividing lines and white space. (**b**) LC3 autophagy marker detection (FITC) by immunofluorescence analysis in MCF-7 cell incubated (Ru) or not (Ctrl) for 48 h with an IC_50_ concentration of the HoThyRu/DOTAP nanosystem. Nuclei are stained in blue (DAPI). In merged images (MERGED), the fluorescent patterns are overlapped. Positive control (Rap) was performed by treatment with 10 µM rapamycin for 48 h. Percentage of LC3-positive MCF-7 cells after the indicated treatments *in vitro* with respect to untreated control cells. ***p < 0.001 *vs*. control cells. (**c**) Quantitation by densitometric analysis of the bands resulting from MCF-7 and MDA-MB-231 cell extracts, plotted in bar graphs as percentage of controls, as indicated. Shown are the average ± SEM values of three independent experiments. The anti-β-actin antibody was used to standardize the amount of proteins in each lane. ***p < 0.001 *vs*. control cells.

Autophagy is a very complex process involving at least 16 proteins that evolves towards the formation of autophagosomes. LC3 protein is the only one known to form a stable association with the membrane of autophagosomes. It is known to exist in two forms: LC3-I, which is found in the cytoplasm and LC3-II, which is membrane-bound and is converted from LC3-I to initiate the formation and lengthening of the autophagosome^34,36^. In this frame, detection of LC3 expression by immunoblot analysis is a useful biomarker to perceive possible autophagy activation. As shown in Fig. 5a, protein samples from MCF-7 and MDA-MB-231 provide evidence of a significant increase in both forms of LC3 protein following exposure *in vitro* to HoThyRu/DOTAP liposomes for 48 and 72 h (quantization in 5c). Antibody for LC3 detection recognizes both the forms: LC3-II differs from LC3-I only in the fact it is covalently modified with lipid extensions and has undergone removal of a short amino acid sequence^44^. This result is consistent with the previously reported autophagy flux detection, as well as with autolysosomes occurrence in late steps of autophagic cell death process. Moreover, this outcome is in line with our recent findings about the ability of Ru-containing cationic nanosystems to activate autophagic cell death pathways^9^.

According to the immunoblot analysis, fluorescent images in Fig. 5b of MCF-7 cells treated with HoThyRu/DOTAP and stained for LC3 demonstrate an intense protein immunoreactivity likely localized into autophagic vacuoles in the cell cytoplasm. LC3 either appears as a diffuse cytosolic signal matching to cytosolic LC3-I or as more punctuate staining autophagosome-associated LC3-II. As for the autophagy flow monitoring experiments, we used rapamycin as autophagy inducer to stimulate autophagosome-associated LC3 expression under the same experimental conditions described above (10 µM, 48 h). MCF-7 incubation with rapamycin or HoThyRu/DOTAP resulted in a significant increase in punctuate staining, as also shown by the percentage of LC3-positive MCF-7 cells with respect to untreated controls. The most intense fluorescent signals are observed in the proximity of nuclei counterstained with DAPI, indicating the formation of autophagosomes.

Finally, we studied the expression of the regulatory protein Beclin 1, that has a central role in autophagy and is frequently decreased in cancer^37,38,45^. Immunoblot experiments (Fig. 5a) performed on MCF-7 and MDA-MB-231 show no significant changes in protein content following exposure to the HoThyRu/DOTAP liposomes with respect to untreated cells. With regard to the antiapoptotic Bcl‐2 family proteins, they are frequently overexpressed in cancers and proteins such as Bcl‐2 are also endowed with antiautophagic abilities due to their action as cell survival factors^46^. In line with previous findings, treatment *in vitro* with HoThyRu/DOTAP at its IC_50_ value substantially reverts Bax/Bcl-2 ratio with respect to basal amounts. In fact, expression profile by Western blot analysis demonstrate that, upon these treatments, Bax is significantly increased and Bcl-2 decreased, approximately by the same extent in MCF-7 and MDA-MB-231 cells. As well predisposing cells to apoptosis, Bcl-2 down-regulation can promote the activation of autophagic cell death.

### Anticancer efficacy of HoThyRu/DOTAP nanosystem in BCC xenografts *in vivo*

**Figure 6:**
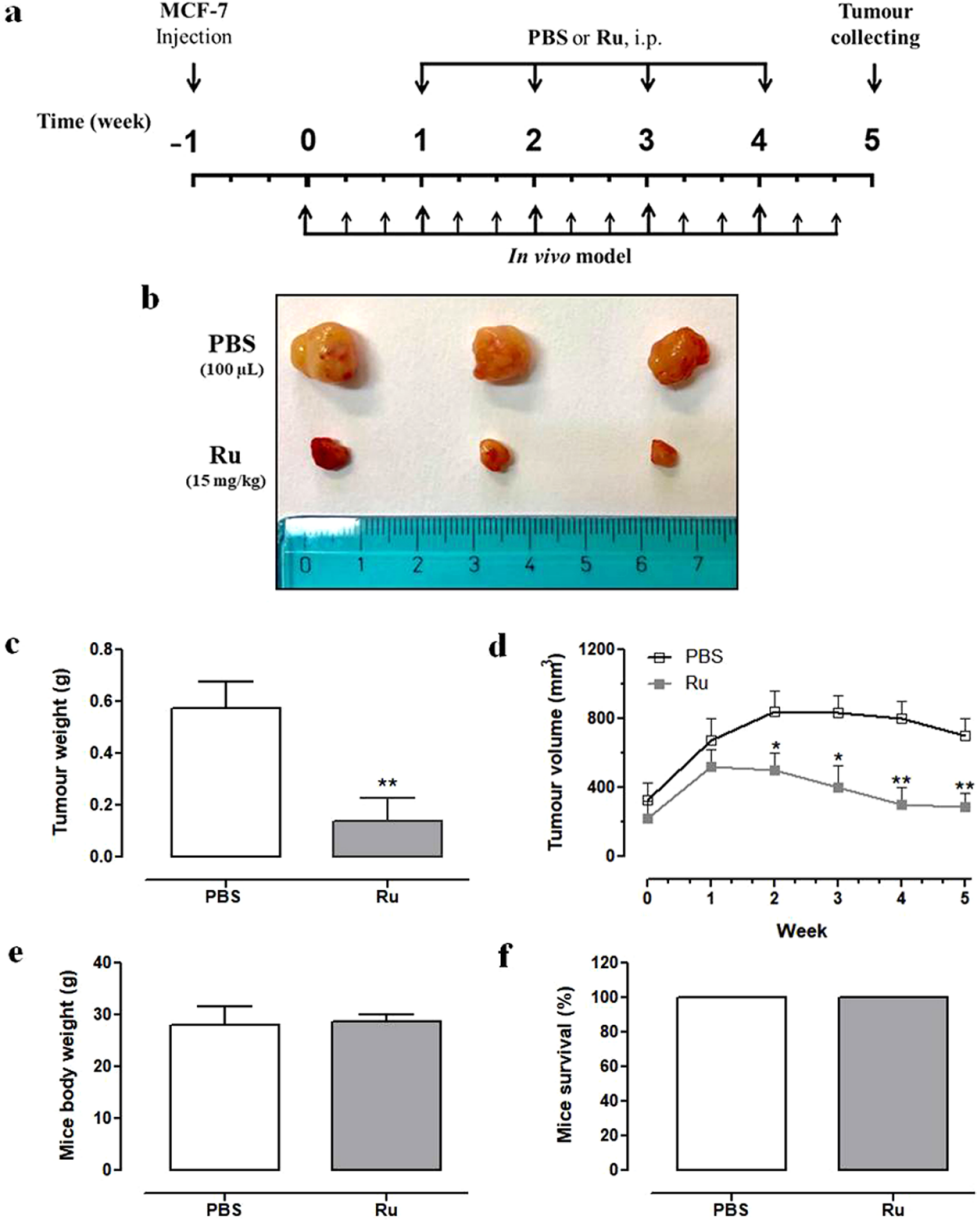
Anticancer effect of the HoThyRu/DOTAP nanosystem *in vivo*. (**a**) Experimental flow chart of the therapeutic strategy with intra-peritoneal (i.p.) HoThyRu/DOTAP (Ru) treatment (15 mg/kg) on athymic nude mice, injected (s.c.) with MCF-7 human breast cancer cells. (**b**) After experiments *in vivo*, tumours were collected, photographed (PBS control group, Ru treated group), (**c**) and tumour weights were determined. (**d**) Tumour size was monitored every week by a caliper, and the volume estimated using the following formula: V = (Length × Width^2^)/2. (**e**) The mice body weight and (**f**) survival (%) were determined. Data are expressed as mean values ± SEM of 1 experiment (*n* = 5 mice per group). *p < 0.05 *vs*. control group; **p < 0.01 *vs*. control group.

At last, to evaluate the animal biological response to systemic administration of HoThyRu/DOTAP nanosystem, as well as its effects on the progression of breast cancer cells *in vivo*, we performed an antitumour study using athymic nude mice bearing human BCC xenografts. As described in the experimental section, MCF-7 cells (5 × 10^6^ cells/mouse) were subcutaneously inoculated into the right flanks of nude mice, and xenograft breast cancers were established after two weeks (Fig. 6a). In depth analysis of tumour growth throughout experimentation shows that HoThyRu/DOTAP significantly inhibits breast cancer cell proliferation in mice (Fig. 6b). Indeed, after the administration of the HoThyRu/DOTAP formulation at 15 mg/kg (i.p.), once a week for 28 days, the weight and volume of tumours were significantly reduced, compared with the control group (Fig. 6c,d). Worth mentioning, mice survival was of 100%, and body weights were not affected by treatments *in vivo* (Fig. 6e,f). In addition, no sign of toxicity in HoThyRu/DOTAP treated animal group was observed. Taken together, these results also suggest that the HoThyRu/DOTAP treatment regimens were well tolerated by mice.

## Discussion

In order to explore new chemotherapeutic strategies to overcome the drawbacks associated with metal-based drug resistance and side effects, we have here examined the nature of the pathways leading to cell death in human models of breast cancer following ruthenotherapy *in vitro*. The main novelty of this study consists in the demonstration of autophagy involvement among the main molecular mechanisms of cell death behind the antiproliferative action of the cationic HoThyRu/DOTAP nanosystem, currently in preclinical evaluation as anticancer drug against human breast carcinoma cells^9,23^. Indeed, a growing body of evidence is implicating autophagy and its regulation in both cancer development and therapy^35–41^. As well, throughout preclinical trials we have verified the efficacy and safety *in vivo* of our Ru-based nanosystems by human breast cancer cells xenografts, proving for the first time that HoThyRu/DOTAP formulation effectively inhibits tumour growth in mice without any detectable evidence of toxicity. Therefore, our results show overall that this Ru-nanosystem induces apoptosis and autophagy *in vitro*, and blocks tumour growth *in vivo*.

Ruthenium-based drugs have provided in the last decades promising outcomes in the context of anticancer drugs development, showing remarkable antiproliferative activity as well as ability to impact on different steps of the metastatic process^13,47^. Essentially designed to mimic platinum-based drugs for targeting DNA and proteins, they position themselves as safe alternative to cisplatin and its congeners in the panorama of new potential anticancer drugs^12,13,18,19,48^. Currently the ruthenium complex NAMI-A, a combinatory therapy by NAMI-A and gemcitabine, and a second Ru-based anticancer agent named KP1019 have entered clinical trials, during which some patients experienced disease stabilization devoid of severe side effects^15–17,49,50^. In the last years preclinical studies have revealed high antiproliferative effects for our suite of ruthenium-containing nanosystems on different cancer lines from human solid tumours, as above all a selected panel of BBC^9,27–31,51^. Apoptosis has hitherto proven to be involved in the control of cancer cell proliferation after Ru-based agents administration^13^. In accordance, we have already demonstrated that the nucleolipidic Ru(III)-complex HoThyRu loaded in both zwitterionic and cationic nanoformulations strongly induces apoptosis activation in breast cancer models. In particular, the most effective formulations we have tested so far, *i*.*e*. cationic nanosystems such as HoThyRu/DOTAP, simultaneously trigger intrinsic and extrinsic apoptosis pathways, which is a non-infrequent condition to address cellular responses to chemotherapy^9^. These outcomes are consistent with former investigations demonstrating that both Ru(II) and Ru(III)-complexes can activate the apoptotic pathways in different cancer cells *in vitro*^13^. Besides apoptosis, here we show that the concomitant activation of autophagy plays a central role in determining the antitumor effect of the cationic HoThyRu/DOTAP nanosystem *in vitro*. Unlike the “naked” AziRu, we have detected both cytosolic and nuclear wide distribution of the active Ru(III) complex after nanosystem application to cells, allowing ruthenium complex to interact with different targets and accounting for its ability to inhibit BBC proliferation. Nucleolipidic nanoformulations co-aggregated with the cationic lipid DOTAP have in fact proved to be particularly effective in the transport of the active ruthenium complex within cancer cells, ensuring a significant drug accumulation in very short time^30,31^. Rapid and massive cell uptake kinetics could be somehow involved in favouring the activation of different cell death pathways. Indeed, large amounts of the Ru(III) complex quickly cross membranes and accumulate in cancer cell, thus having the opportunity to simultaneously interact with different targets, both nuclear and cytosolic. Autophagy itself, as well as autophagy dysregulation, definitely plays important antitumor role in early stages of carcinogenesis, but at the same time protects high proliferative cells against a variety of stressors, acting as an adaptive response to promote growth and progression. In fact, while cancer growth proceeds, cells can be exposed to unfavourable conditions in the tumor microenvironment - *i*.*e*. nutrient shortage and hypoxia, growth factors deficiency and oxidative stress - wherein autophagy induction may sustain cell survival^36,40^. As well, induction of autophagy could be a compensatory response to therapeutic stress, since autophagy inhibitors have been demonstrated in some cases to enhance the efficacy of cytotoxic treatments^52^. Therefore, nonetheless compensatory responses could also be involved in the development of cancer chemoresistence, the role of autophagy in this context remains substantially unknown. On the other hand, such an extensive and sustained autophagy activation - as the one here found in BCC models after treatments by HoThyRu/DOTAP - provides an important contribution in causing cell death. Massive induction of autophagy may hinder cancer cell survival acting as a tumour suppressor factor^53^. Indeed, autophagy potential as therapeutic target has been the subject of several investigations, and anticancer interventions showing the ability to trigger autophagic responses are promising therapeutic approaches^37–41^. The autophagosomal marker LC3-II reflects autophagic activity, so that LC3 monitoring by both immunoblotting and immunofluorescence has become a consistent method for autophagy detection, including autophagic cell death. The activation of this process is always associated with changes in the expression of LC3 protein^44^. In line, we have shown a consistent increase in both forms of LC3 after treatments. Noteworthy, the LC3-phosphatidylethanolamine conjugate (LC3-II), recruited to autophagosomal membranes, was the most increased form^54^. Hence, allowing for the role of LC3 in the autophagic process, HoThyRu/DOTAP is able to induce autophagy activation *in vitro*. As far as other autophagy-related proteins are concerned, Beclin 1-a Bcl-2 interacting protein - is the first identified mammalian gene to mediate autophagy, also having tumour suppressor and antiviral function. This protein is a component of the phosphatidylinositol-3-kinase (PI3K) complex, which is required for autophagosome formation mediating vesicle-trafficking processes, and is thought to play a role in several cellular processes, including tumorigenesis and apoptosis^45^. The expression of Beclin 1 is in fact frequently decreased in malignant breast epithelial cells. Based on these observations, it is speculated that Beclin 1 may work through induction of autophagy to negatively regulate tumor progression. In fact, the autophagy-promoting activity of Beclin 1 in MCF-7 cells has been associated with inhibition of cellular proliferation^55^. Interestingly, we did not observe any decrease in its expression after treatments with HoThyRu/DOTAP in BCC models. Given the importance of Beclin-1 for autophagy, the maintenance of basal protein amounts during ruthenotherapy could be positively correlated with the activation of autophagic process^56^. This is another significant feature considering that some recent experimental evidence showed a lack of autophagic pathways following total Beclin 1 depletion induced by different conditions, including chemotherapeutic interventions^57^. Excessive autophagy is associated with cell death, as well as with death pathways other than this depending on cell types - an aspect to be taken into account as ruthenotherapy additionally activates apoptosis^58,59^. In particular, autophagy and apoptosis may be triggered by common upstream signals, resulting in combined autophagy and apoptosis. Recent studies have suggested possible molecular mechanisms for crosstalk between autophagy and apoptosis, by interaction and cooperation between proteins such as Bcl-2 family proteins and Beclin 1, and regulatory proteins other than Beclin 1^60^. Since the oncogenic Bcl-2 protein inhibits apoptosis by binding Bax and other factors, as well as autophagy by interacting with Beclin 1, its down-regulation found in both MCF-7 and MDA-MB-231 as a consequence of the HoThyRu/DOTAP treatment can predispose BCC to both apoptosis and autophagy. This is also consistent with our former findings^9^. However, the crosstalk between apoptosis and autophagy is complex and remains to be defined, given its prospective relevance for future improved cancer treatments. The discovery of interconnections among different cell death mediators and signaling pathways will in fact offer novel opportunities for targeted cancer therapies^61^. Accordingly, our preclinical investigations suggest that an original Ru(III)-dependent approach, based on both apoptosis activation and sustained autophagy induction, could provide new therapeutic options for breast cancer treatment, including the highly aggressive triple-negative (TNBC) subtype. Potential agents able to trigger multiple biological responses are attracting more and more interest in the field of promising therapeutic strategies to address chemoresistance and tumour spread, especially if coupled to nanoparticles utilization as the means of targeted delivery while avoiding or reducing undesired side-effects^62,63^. In-depth studies on the interplay between autophagy and apoptosis are necessary and likely to have critical implications for further developments of novel multi-target Ru-based agents for cancer chemotherapy.

## Methods

### HoThyRu/DOTAP and HoThyDansRu/DOTAP liposome preparation

The here investigated ruthenium(III) complex, named HoThyRu, was prepared by reacting in stoichiometric amounts the starting nucleolipid HoThy (Fig. S2) with the Ru complex [*trans*-RuCl_4_(DMSO)_2_]^−^Na^+^ following a previously described procedure^27,30,64^. A similar procedure was followed to prepare the fluorescently labelled complex HoThyDansRu starting from the nucleolipid HoThyDans (Fig. S3), as previously described^30^. The desired nucleolipidic Ru(III) complexes were obtained in a pure form, as confirmed by TLC and ESI-MS analysis, and almost quantitative yields. The lipid formulations of HoThyRu and HoThyDansRu in DOTAP were prepared as previously reported and characterized by DLS analysis^28,30^.

### Cell cultures

Epithelial-like type human breast adenocarcinoma cells MCF-7 (Endocrine-Responsive, ER) and MDA-MB-231 (Triple-Negative Breast Cancer, TNBC) were grown in DMEM (Invitrogen, Paisley, UK) supplemented with 10% fetal bovine serum (FBS, Cambrex, Verviers, Belgium), L-glutamine (2 mM, Sigma, Milan, Italy), penicillin (100 units/ml, Sigma) and streptomycin (100 μg/ml, Sigma), and cultured in a humidified 5% carbon dioxide atmosphere at 37 °C. MCF 10A (kindly provided by Valeria Cicatiello, Italian National Research Council (CNR), Institute of Genetics and Biophysics, Naples, Italy), a non-tumourigenic mammary gland epithelial cell line^65^, was maintained in DMEM (Invitrogen, Carlsbad, CA) supplemented with 5% horse serum (Invitrogen), 500 ng/ml hydrocortisone (Sigma-Aldrich, St. Louis, MO), 100 ng/ml cholera toxin (Sigma-Aldrich), 10 μg/ml insulin (Invitrogen), 20 ng/ml epidermal growth factor (EGF, Sigma-Aldrich), penicillin (100 units/ml, Sigma) and streptomycin (100 μg/ml, Sigma), and cultured in a humidified 5% carbon dioxide atmosphere at 37 °C, according to ATCC recommendations.

### Bioscreens *in vitro*

The cytotoxic activity of the ruthenium-containing nucleolipidic nanosystem HoThyRu/DOTAP was investigated through the estimation of a “cell survival index”, arising from the combination of cell viability evaluation with cell counting, as previously reported^9,31^. The cell survival index is calculated as the arithmetic mean between the percentage values derived from the MTT assay and the automated cell count. Cells were inoculated in 96-microwell culture plates at a density of 10^4^ cells/well and allowed growing for 24 h. The medium was then replaced with fresh medium and cells were treated for additional 48 h with a range of concentrations (1 → 250 μM) of HoThyRu/DOTAP liposomes. Using the same experimental procedure, cell cultures were also incubated with ruthenium-free HoThy/DOTAP liposomes as negative controls, as well as with cisplatin (*c*DDP) - a positive control for cytotoxic effects. Cell viability was evaluated using the MTT assay procedure^66^. Cell number was determined by TC20 automated cell counter (Bio-Rad, Milan, Italy), providing an accurate and reproducible total count of cells and a live/dead ratio in one step by a specific dye (trypan blue) exclusion assay. The calculation of the concentration required to inhibit the net increase in the cell number and viability by 50% (IC_50_) is based on plots of data (*n* = 6 for each experiment) and repeated five times (total *n* = 30). IC_50_ values were calculated from a dose response curve by nonlinear regression using a curve fitting program, GraphPad Prism 5.0, and are expressed as mean values ± SEM (*n* = 30) of five independent experiments.

### Subcellular fractionation

MCF-7 and MDA-MB-231 cells were grown on standard 100 mm culture dishes by plating 8 × 10^5^ cells. After 24 h of growth, the cells were incubated with 100 µM of AziRu or with 100 µM of HoThyRu/DOTAP liposome for 24 h under the same experimental conditions described for bioscreen assays. At the end of the treatment, the culture medium was collected and the cells were enzymatically harvested by trypsine, then centrifuged at RT for 3 min at 300 × *g*. The cell pellets obtained were resuspended in 500 μl of a solution I (10 mM HEPES pH 7.9, 10 mM KCl, 0.1 mM MgCl_2_, 0.1 mM EDTA, 0.1 mM DTT, Protease Inhibitor Cocktail) and centrifuged at 15,000 × *g* for 10 min at 4 °C. The supernatant, representing the cytosolic fraction, was separated from the pellet which in turn contained the nuclear and mitochondrial fraction^67^. Furthermore, the pellets were washed 3 times with the solution I and 200 µl of lysis buffer (10 mM HEPES, 3 mM MgCl_2_, 40 mM KCl, 5% glycerol, 1 mM DTT, 0.2% NP40) was added and incubated for 30 min in ice. After centrifugation at 4 °C for 30 min at 500 × *g*, the pellets containing the nuclear fraction were obtained. To obtain the purified DNA fraction, the pellets were suspended in DNA lysis buffer (50 mM Tris-HCl, pH 8.0, 0.5 mM EDTA, 100 mM NaCl, 1% SDS, 0.5 mg/mL proteinase K) and incubated at 50 °C for 1 h. After incubation, 10 mg/mL RNase was added to the lysates and incubated for 1 h at 50 °C. DNA was precipitated with NaOAc pH 5.2 and ice cold 100% EtOH and then centrifuged at 14000 × *g* for 10 min. The pellets were dissolved in TE buffer (10 mM Tris-HCl, pH 8.0, 1 mM EDTA). Aliquots of culture medium, cellular pellet, cytosolic fraction, nuclear fraction and DNA sample were analyzed by inductively coupled plasma-mass spectrometry (ICP-MS) to determine the ruthenium amounts in each sample.

### Inductively coupled plasma mass spectrometry (ICP-MS)

Inductively Coupled Plasma–Mass Spectrometry (ICP-MS) was used for the rapid and highly sensitive determination of ruthenium concentrations in cells treated with the HoThyRu/DOTAP nanosystem, using those treated with AziRu as control. Samples obtained by subcellular fractionation were subjected to oxidative acid digestion with a mixture of 69% nitric acid and 30% v/v hydrogen peroxide in 8:1 ratio, using high temperature and pressure, under a microwave assisted process. A proper dilution was made and the suspension obtained for each sample was introduced to the plasma. The mineralized samples were recovered with ultrapure water and filtered using 0.45 µm filters. The determination of ruthenium was carried out on Inductively Coupled Plasma Mass Spectrometry (ICP-MS) instrument Aurora M90 Bruker. The quantitative analysis was carried out using the external calibration curve method. In the analyzed fractions, the ruthenium content is expressed as percentage of the total ruthenium administered during incubation *in vitro*.

### Immunostaining, confocal and fluorescent microscopy

With reference to the analysis of the cell internalization process, the fluorescent dansyl-labeled nucleolipidic complex HoThyDansRu (above described) has been used. Non auto-fluorescent poly-D-Lysine coated sterile glass coverslips (neuVitro, El Monte, CA, USA) were placed in standard sterile plastic 24-well plates and human MCF-7 breast adenocarcinoma cells were seeded at a density of 4 × 10^4^/well and allowed for growth. Cells were then exposed or not to 100 µM of the HoThyDansRu/DOTAP nanosystem for 0.5, 1, 2, 4, and 6 h, under the same experimental conditions described for bioscreen *in vitro*. After treatments, unassociated liposomes were removed by PBS washing (three times) and cells were fixed for 20 min with a 3% (w/v) paraformaldehyde (PFA) solution, and permeabilized for 10 min with 0.1% (w/v) Triton X-100 in phosphate-buffered saline (PBS) at RT. To prevent nonspecific interactions of antibodies, cells were treated for 2 h in 5% bovine albumin serum (BSA) in PBS. Immunostaining was carried out by incubation with Alexa Fluor 647 mouse anti-human CD324 (E-Cadherin) monoclonal antibody (Biolegend, CA, USA), which recognizes the extracellular domain of human E-Cadherin, a 120-kDa transmembrane glycoprotein localized in the adherens junctions of epithelial cells. DAPI/Moviol Pro Long Diamond Antifade Mountant with DAPI (Invitrogen/Thermo Fisher Scientific, Waltham, MA, USA) was used as nuclear stain. The coverslip from each well was mounted onto a glass microslide with 80% fluorescence-free glycerol mounting medium. The analyses were performed with a Zeiss LSM 510 microscope equipped with a plan-apochromat objective X 63 (NA 1.4) in oil immersion. For LC3 immunostaining, sterile coverslips were placed in six-well plates and MCF-7 cells were seeded at a concentration of 2 × 10^4^ per ml. Following a growth period of 24 h at 37 °C in DMEM containing 10% FBS, the medium was replaced with fresh medium and cultures were treated or not with 100 µM of HoThyDansRu/DOTAP for 48 h. After treatments *in vitro*, cells were washed three times with PBS, fixed with 4% PFA for 15 min, and permeabilized with Triton X-100 (0.1%) for 90 min. Cells were then incubated with 1:1000 rabbit polyclonal primary antibody to human LC3B (Novus Biologicals, LLC, Littleton, CO, USA) for 90 min at room temperature. Next, cells were washed with PBD and treated for 90′ min in the dark at RT with a goat anti-rabbit IgG-FITC as secondary antibody labeled with fluorescein isothiocyanate (FITC) for immunofluorescence staining. Finally, MCF-7 cells were examined by a fluorescent microscope (Leica Microsystems GmbH, Wetzlar, Germany) to visualize DAPI (345/661 nm) as nuclear stain and fluorescent immunocomplexes (557/571 nm). Images were taken by an AxioCam HRc video-camera (Zeiss) connected to an Axioplan fluorescence microscope (Zeiss) using the AxioVision 3.1 software.

### Autophagy flux detection

Induction of the autophagic cell death pathway was investigated by an “Autophagy Detection Kit” (ab 139484, Abcam, Cambridge, USA), which measures autophagic vacuoles and monitors autophagic flux in live cells using a novel dye, selectively labeling autophagic vacuoles. The dye has been optimized through the identification of titratable functional moieties that allow for minimal staining of lysosomes while exhibiting bright fluorescence upon incorporation into pre-autophagosomes, autophagosomes, and autolysosomes (autophagolysosomes). In brief, MCF-7 and MDA-MB-231 cells were cultured on 96-microwell culture plates, and allowed growing until reaching ~70% level of confluence; then the medium was carefully replaced with fresh medium and cells were incubated for 48 and 72 h with 100 μM of HoThyRu/DOTAP liposomes. According to the supplier’s instructions, positive control cells were pre-treated with 10 µM rapamycin as autophagy inducer for 48 h (timing and concentration experimentally determined). Negative control cells were treated with DMSO as vehicle and/or with the complete culture medium (to reconstitute or dilute the inducer) for an equal length of time under similar conditions. After treatments, the medium with the testing reagents and positive control was removed, cells were washed twice with 1X Assay buffer and then treated with 100 μl of Microscopy Dual Detection Reagent to cover each sample of cell monolayer. Samples were protected from light and incubated for 30 min at 37 °C. Finally, cells were carefully washed with 100 μl of 1X Assay Buffer and excess buffer was removed before placing microplates on microscope slide. Stained cells were analyzed by wide-field fluorescence (RiS™ Digital Cell Imaging System from Twin Helix) at 40× magnification [objective TC PlanFluor 40× (NA 0.6, WD 2.9)] using a standard FITC filter set for imaging the autophagic signal (EGFP fluorescent filter cubes Ex470/30, Em530/50) and the nuclear signal using a DAPI filter set for nuclear signal (DAPI fluorescent filter cubes Ex375/28, Em460/50). The percentage of positive cells in the test was determined by fluorescence microscopy, according to the following criteria: the Green Detection Reagent-positive cells were determined by counting the number of cells with green signal in comparison to the number of cells with only DAPI fluorescence. Optimal parameter settings were found using Green Detection Reagent-positive controls.

### Preparation of cellular extracts

MCF-7 and MDA-MB-231 cells were cultured in standard 60 mm culture dishes by plating 5 × 10^5^ cells. Behind reaching the subconfluence, cells were incubated for 48 and 72 h with IC_50_ concentrations of the HoThyRu/DOTAP formulation under the same experimental conditions herein described. After treatments, cells were washed and collected by scraping with PBS containing 1 mM EDTA and low-speed centrifugation. Cell pellets were then lysed at 4 °C for 30 min in a buffer containing 20 mM Tris–HCl, pH 7.4, 150 mM NaCl, 5 mM EDTA, 5% (v/v) glycerol, 10 mM NP-40 and protease inhibitor tablets (Roche). The supernatant fraction was obtained by centrifugation at 15,000 × *g* for 10 min at 4 °C and then stored at −80 °C^68^. Protein concentration was determined by the Bio-Rad protein assay (Bio-Rad, Milan, Italy).

### Western blot analysis

Samples containing 30–50 µg of proteins were loaded on 10% SDS–PAGE and transferred to nitrocellulose membranes^69^. After blocking at RT in milk buffer [1 × PBS, 5–10% (w/v) non-fat dry milk, 0.2% (v/v) Tween-20], the membranes were incubated at 4 °C overnight with 1:1000 rabbit polyclonal antibody to human LC3B (Novus Biologicals), and with 1:100 mouse monoclonal antibody to human BECN1(E-8) (Santa Cruz Biotechnology, Santa Cruz), with 1:500 rabbit polyclonal antibody to human Bcl-2 (Santa Cruz Biotechnology) and 1:250 rabbit polyclonal antibody to human Bax (Santa Cruz Biotechnology). Subsequently, the membranes were incubated with peroxidase-conjugated goat anti-rabbit IgG, or with peroxidase-conjugated goat anti-mouse IgG + IgM (all the secondary antibodies were purchased from Jackson ImmunoResearch Laboratories). The resulting immunocomplexes were visualized by the ECL chemoluminescence method (ECL, Amersham Biosciences Little Chalfont, Buckinghamshire, UK) and analyzed by an imaging system (ImageQuantTM400, GE Healthcare Life Sciences). Densitometric analysis was carried out using the GS-800 imaging densitometer (Bio-Rad)^70^. Normalization of results was ensured by incubating the nitrocellulose membranes in parallel with the β-actin antibody (Sigma-Aldrich).

### Tumor xenograft in nude mice

Animals (4-week old female athymic nude Foxn1nu mice, 25 g of weight) were purchased from Envigo RMS (Udine, Italy) and kept in an animal care facility under controlled temperature, humidity and on a 12:12 h light-dark cycle, with *ad libitum* access to water and standard laboratory chow diet. Tumours were established by subcutaneous injection (s.c.) of 5 × 10^6^ breast adenocarcinoma MCF-7 cells, 1:3 mixed in Matrigel® Matrix (Growth Factor Reduced, Corning, Bedford, U.S.A.), into the right flank of each mice. Mice were randomly assigned to one of the two experimental groups (control group and treated group, *n* = 5 each). Treatments began two weeks post tumour implant with intraperitoneal injection (i.p.) of HoThyRu/DOTAP formulation (15 mg/kg), administered to mice 1 time/week for 28 days. Control group was treated with 100 µl PBS (1X, Gibco). Tumour volumes, monitored and recorded every two days, were calculated using the formula V = (Lenght × Width^2^)/2. As well, mice body weights were recorded every two days by MS-Analytical and Precision Balance (Mettler Toledo). At the end of treatments, animals were sacrificed, and tumours collected and carefully weighed. The mice survival data, tumour volumes and weights were plotted using Graph-Pad Prism software (version 6.01), and *p* values were calculated. All experimental procedures were carried out in compliance with the international and national law and policies (EU Directive 2010/63/EU for animal experiments, ARRIVE guidelines and the Basel declaration including the 3R concept) and approved by Italian Ministry of Health (n. 354/2015-PR). All procedures were carried out to minimize the number of animals used (*n* = 5 per group) and their suffering.

### Statistical data analysis

All data were presented as mean values ± SEM. The statistical analysis was performed using Graph-Pad Prism (version 6.01, Graph-Pad software Inc., San Diego, CA) and ANOVA test for multiple comparisons was performed followed by Bonferroni’s test.

## Supplementary information

Molecular structures of the Ru(III) complexes NAMI-A (a), KP1019 (b) and AziRu (c).

Molecular structure of the nucleolipid HoThy.

Molecular structure of the nucleolipid HoThyDans.

Supplementary Information
