# Supplementary material for: Exploring cellular uptake, accumulation and mechanism of action of a cationic Ru-based nanosystem in human preclinical models of breast cancer
Source: Sci Rep. 2019 May 7;9:7006. doi: 10.1038/s41598-019-43411-3 (PMC6505035; doi:10.1038/s41598-019-43411-3)
Supplement: Supplementary file 3 — Molecular structure of the nucleolipid HoThyDans. [file 41598_2019_43411_MOESM3_ESM.pdf]

Exploring cellular uptake, accumulation and mechanism of action of a cationic Ru-based nanosystem in human preclinical models of breast cancer

Marialuisa Piccolo[1,#], Gabriella Misso[2,#], Maria Grazia Ferraro[1], Claudia Riccardi[3], Antonella Capuozzo[1], Mayra Rachele Zarone[2], Francesco Maione[1], Marco Trifuoggi[3], Paola Stiuso[2], Gerardino D'Errico[3,4] ,Michele Caraglia[2], Luigi Paduano[3,4,*], Daniela Montesarchio[3,*], Carlo Irace[1,°,*] and Rita Santamaria[1,°]

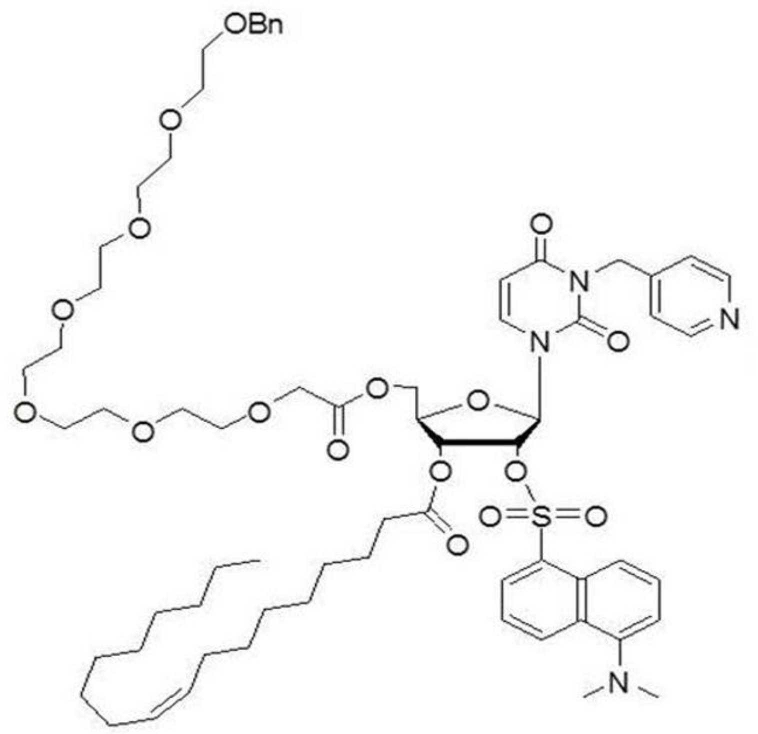

Fig. S3. Molecular structure of the nucleolipid HoThyDans.
